# Permeation of Silver Sulfadiazine Into TEMPO-Oxidized Bacterial Cellulose as an Antibacterial Agent

**DOI:** 10.3389/fbioe.2020.616467

**Published:** 2021-01-28

**Authors:** Shahia Khattak, Xiao-Tong Qin, Fazli Wahid, Long-Hui Huang, Yan-Yan Xie, Shi-Ru Jia, Cheng Zhong

**Affiliations:** ^1^State Key Laboratory of Food Nutrition & Safety, Tianjin University of Science & Technology, Tianjin, China; ^2^Key Laboratory of Industrial Fermentation Microbiology, (Ministry of Education), Tianjin University of Science & Technology, Tianjin, China

**Keywords:** silver sulfadiazine, bacterial cellulose, TEMPO-oxidation, antibacterial activity, nanocomposites

## Abstract

Surface oxidation of bacterial cellulose (BC) was done with the TEMPO-mediated oxidation mechanism system. After that, TEMPO-oxidized bacterial cellulose (TOBC) was impregnated with silver sulfadiazine (AgSD) to prepare nanocomposite membranes. Fourier transform infrared spectroscopy (FTIR) was carried out to determine the existence of aldehyde groups on BC nanofibers and X-ray diffraction (XRD) demonstrated the degree of crystallinity. FESEM analysis revealed the impregnation of AgSD nanoparticles at TOBC nanocomposites with the average diameter size ranging from 11 nm to 17.5 nm. The sample OBCS_3_ showed higher antibacterial activity against *Staphylococcus aureus, Pseudomonas aeruginosa*, and *Escherichia coli* by the disc diffusion method. The results showed AgSD content, dependent antibacterial activity against all tested bacteria, and degree of crystallinity increases with TOBC and AgSD. The main advantage of the applications of TEMPO-mediated oxidation to BC nanofibers is that the crystallinity of BC nanofibers is unchanged and increased after the oxidation. Also enhanced the reactivity of BC as it is one of the most promising method for cellulose fabrication and functionalization. We believe that the novel composite membrane could be a potential candidate for biomedical applications like wound dressing, BC scaffold, and tissue engineering.

## Introduction

Bacterial cellulose (BC) is one of the most promising biopolymers due to its environmentally friendly nature (Shao et al., 2017), which can be used for treatment of various bacterial infections after chemical modification (Zmejkoski et al., 2018). Bacterial infections being a chief health hazard necessitates antibacterial strategies, among which antibiotics are the most frequently used treatment (Percival et al., 2015; Qian et al., 2017). However, drug-resistant pathogens have forced researchers to explore novel antibacterial agents (Almeida et al., 2014; Ge et al., 2014). BC has several advantages over plant-derived cellulose, including high purity (Reiniati et al., 2017), high crystallinity, high elasticity (Wang et al., 2016), thermal stability, high degree of polymerization (Lin et al., 2013), excellent permeability, high porosity, water content, and high mechanical strength (Moniri et al., 2017). In spite of the several advantages, the significant drawback of the BC is that it lacks antibacterial property (Sulaeva et al., 2015). Modified BC is a splendid cure to all these failings of conventional antibiotics. Various treatments can be used to improve the properties of BC (Fijałkowski et al., 2017). Such modification could improve/boost the properties of BC, for the applications in food industry as well as biomedical field (Habibi, 2014; Paximada et al., 2016). However, to obtain derivatives with innovative features, surface modification of BC (hydroxyl group on the surface of the BC) is an appealing strategy (Zmejkoski et al., 2018).

**Figure 1:**
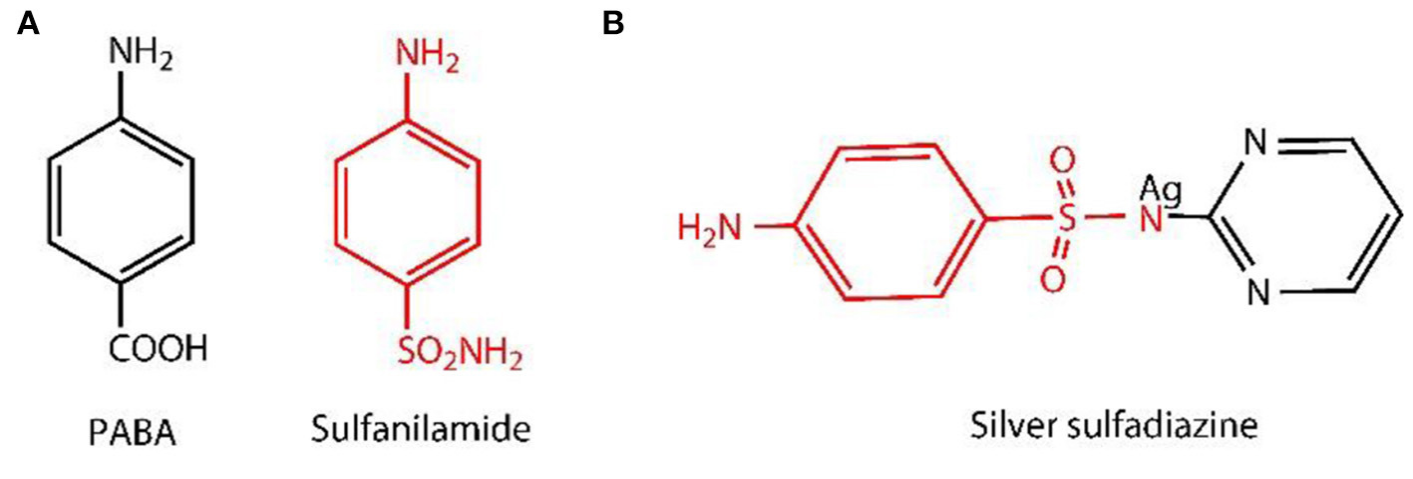
Para-aminobenzoic acid (PABA) and structural analog of PABA (sulfanilamide) **(A)** and chemical structure of AgSD **(B)**.

**Figure 2:**
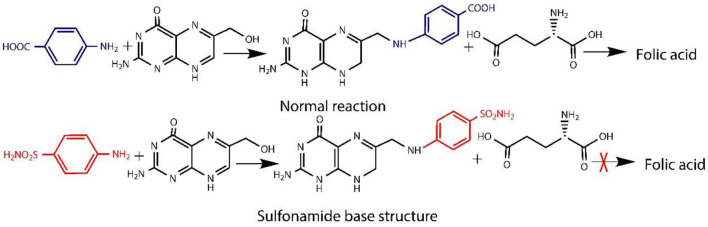
Synthesis of Folic acid and sulfonamide action site.

As the modification at C2 and C3, of the BC, usually leads to the decomposition of BC oligomers in case of using periodate oxidation (Saito and Isogai, 2004), so, in order to maintain the mechanical properties of BC, it is necessary to modify the hydroxyl group at the main surface only, i.e., selective oxidation (Lee et al., 2012). The commonly used method is the oxidation under alkaline conditions with TEMPO used in combination with NaBr/NaOCl (Saito and Isogai, 2004), because it binds the aldehydic (-CHO) and carboxylic (-COOH) functional groups obtained from the hydroxylic group present at the C6 position of BC. The surface modification with carboxylic groups promotes the decomposition of hydrogen bonds, which improve the accessibility of macromolecules and enhanced the reactivity (Okita et al., 2010). Also, the speed of reaction is high and is one of the most promising method for cellulose fabrication and functionalization (Isogai et al., 2011). TEMPO-mediated oxidation only increases the uniformity of accessibility of the reactive BC carboxylate (Lai et al., 2013). It did not induce antibacterial activity, which restricts/bounds the possibility of applications in the areas of biomedical applications. So, in order to solve this problem, some researchers have added Ag and Ag nanocomposites as an antibacterial agent (e.g., Ag, ZnO, and graphene oxide) in BC matrix (Fortunati et al., 2014; Liu et al., 2017; Khattak et al., 2019). Among them, Ag with sulfadiazine, i.e., AgSD, has been widely used as an antibacterial agent for topical treatment for decades (Atiyeh et al., 2007; Muangman et al., 2010). It also showed broader activity spectrum against *Pseudomonas aeruginosa, Escherichia coli*, and *Staphylococcus aureus* by disrupting the cell membrane (Hoffmann, 1984) and inhibiting the DNA replication (Aguzzi et al., 2014). Antibacterial activity develops by the degradation of AgSD into sulfadiazine and Ag ions (Fox and Modak, 1974; Fox, 1975). The Ag ion of the AgSD interrupts the triphosphate (ATP) synthesis (Liu et al., 2019), whereas sulfadiazine inhibits the synthesis of folic acid (Cook and Turner, 1975; Wei et al., 2011), because of the structural analog of p-aminobenzoic acid (PABA) (Baenziger and Struss, 1976) as shown in the example (Figure 1A). The chemical structure of AgSD is displayed in Figure 1B. Folic acid plays an essential role in the growth and reproduction of bacteria (Craig and Stitzel, 2004; Tacic et al., 2017). Folic acid synthesis and mode of action of sulfonamides (sulfadiazine) are schematically presented (Figure 2).

TEMPO oxidation enhances the reactivity of BC due to which the silver nanocomposites showed excellent antibacterial activity because the extensive surface morphology provides better contact with microorganisms (Tacic et al., 2017). Also, for the synthesis of silver nanoparticles, Ifuku et al. (2009) use the TEMPO-mediated oxidation system to prepare the TOBC pellicle as a reaction template. Recently, a conjugated highly porous antimicrobial dressing was developed by Mohseni et al. (2019), loaded with silver sulfadiazine, and its antibacterial activity and biocompatibility were evaluated. Silver-carboxylated nanocellulose was prepared from bagasse waste in treatment with ammonium persulfate using a facile and green photochemical approach (Caschera et al., 2020). In another study, the porous cellulose particles with solvent-releasing method, exhibiting high catalytic ability, were prepared and evaluated (Fujii et al., 2020). Garza-Cervantes and his co-workers synthesized a novel silver-containing biocomposite using green methodology (Garza-Cervantes et al., 2020). Wang and his co-workers developed an electroactive regenerated hydrogel with significant exhibited higher electrical conductivity (Wang et al., 2020). In another reported study, HEMA-based skin repair hydrogel was prepared with BC by polymerization (Di et al., 2017). Furthermore, thermally stable electrically conductive polyaniline-based BC biosensor nanotubes were prepared by Jasim et al. (2017). Sajjad and his co-workers also developed curcumin-based BC nanocomposites as wound dressing (Sajjad et al., 2020). Biopolymers like BC and chitosan were used to prepare scaffolds (Ul-Islam et al., 2019). Along with the above cited literature, silver nanoparticles have also been used in several applications incorporated with polymers like polyvinylpyrrolidone (PVP), polymethyl methacrylate (PMMA), zein, polyacrylonitrile (PAN), chitosan, 3D printing, and others as well (GhavamiNejad et al., 2015; Yang C. H. et al., 2016; Ullah et al., 2019). The commonly used modification methods include nanoparticle coating and metal oxide modification (Smith et al., 2017; Yao et al., 2018). Therefore, BC was considered to be an ideal matrix for these modifications (Martínez-Sanz et al., 2012; Yang et al., 2012; McCarthy et al., 2019). In the present study, TEMPO oxidation was used to introduce the carbonyl group (carboxylate) to BC, and it was further composited with AgSD particles. The main advantage of the TEMPO-mediated oxidation is the retained crystallinity of BC after oxidation and more reactivity while silver sulfadiazine (AgSD) makes it antibacterial. We believe that the composite membrane will find promising applications in the biomedical field like wound dressing, BC scaffolding, and tissue engineering.

## Materials and Methods

### Materials

Silver sulfadiazine with molecular weight 357.14 g/mol was obtained from Meryer (Shanghai) Chemical Technology Co., Ltd. (Shanghai, China). Tryptone and yeast extract were obtained from Oxoid Ltd. (United Kingdom). Agar powder was purchased from Beijing Solarbio Science and Technology Co., Ltd. All other chemicals such as CH_3_COOH, NaOH, and Na_2_HPO_4_ were of analytical grade.

### Methods

#### Preparation of BC Membrane and Incubation Conditions

Microorganisms *Komagataeibacter xylinus* (*K. xylinus*) (CGMCC No. 2955) were evaluated/screened by our group (Zhong et al., 2013). BC films were prepared by the previously described method (Zhong et al., 2014; Yang X. N. et al., 2016). In brief, *K*. *xylinus* was cultured in a culture medium consisting of glucose (25.0 g/L), peptone (10 g/L), yeast extract (7.5 g/L), and disodium phosphate (10.0 g/L). The cultured medium pH was kept/adjusted to 6.0. The bacteria were inoculated into culture medium in a flask. The incubation was carried out at a temperature of 30°C and with a speed of 160 rpm for 24 h in a shaker. With a ratio of 8% (v/v), the cell suspension was inoculated into different culture medium and kept in incubation for 7 days at 30°C (Zhong et al., 2013). The BC membranes obtained were kept in alkaline solution (0.1 M NaOH) to remove the impurities. Membranes were washed with distilled water until neutral pH and stored for further use.

### TEMPO-Mediated Oxidation of BC

BC was treated with a homogenizer (120 W) (Hualeda Instrument Co., Ltd, Beijing, China) until formation of the homogeneous slurry/pulp. In deionized water (100 ml), sample (1 g dry weight) of BC was suspended and stirred on a magnetic stirrer to form the homogeneous suspension of BC to properly oxidize it. In 20 ml of deionized water, sodium bromide (NaBr) (0.1 g) and TEMPO (0.016 g) were dissolved in order to form the TEMPO medium. This TEMPO-mediated solution was added to BC suspension with magnetic stirring. To initiate TEMPO-mediated oxidation, NaClO solution was added 10% (0.5 mmol/g) to the TEMPO-mediated BC suspension at room temperature. The pH of the solution was maintained between 10.5 and 11.0 with the addition of 0.5 mol/L NaOH solution. After 12 h, the oxidation was quenched by the addition of ethanol (C_2_H_5_OH) (5 ml). The oxidized BC was collected by centrifugation at 1000 rpm for 30 min, and with deionized water, it was washed thoroughly (Habibi et al., 2006; Okita et al., 2010). After washing, the precipitates of oxidized BC (TOBC) were stored at 4°C for further use (experiments).

### Preparation of TOBC–AgSD Nanocomposites

The method of preparing TOBC–AgSD was described in literature with slight modification (Shao et al., 2016). To prepare the TOBC–AgSD composite, AgSD was dissolved in distilled water at room temperature and stirred with a magnetic stirrer the whole day and then sonicated for 90 min to form a homogeneous solution. AgSD was added into the TOBC dispersions and mixed for 1 h at magnetic stirring. The weight ratio of AgSD to TOBC was controlled to be 0.1 wt.%, 0.2 wt.%, and 0.3 wt.% (marked as OBCS_1_, OBCS_2_, and OBCS_3_, respectively). Then, the mixture was treated by ultrasonication for 20 min. The weight of the TOBC (known) is kept constant. After the formation of AgSD–TOBC nanocomposites, samples were filtered and dried in the oven at 40–50°C.

### Characterization

The FT-IR spectra of pure BC, TOBC, and TOBC–AgSD nanocomposites were measured using a Nicolet IS50 FT-IR spectrometer with a wavenumber ranging from 4000 to 400 cm^−1^. XRD patterns of TOBC films and TOBC–AgSD nanocomposites were obtained using a Shimadzu XRD-6100 X-ray diffractometer at 40 kV with a scan range of 5–40° and a scan speed of 5°/min. The morphology of TOBC–AgSD nanocomposites was examined by FESEM. The elemental composition of AgSD was confirmed by EDS [energy-dispersive X-ray (EDX) analysis by EDAX].

### Crystallinity Analysis

The degree of crystallinity (*CrI*) has been calculated by the reported method with slight modification (Pelegrini et al., 2019). Briefly, *CrI* is calculated from the ratio of the area of all crystalline peaks to the total area. XRD spectrum is used to calculate the *CrI*, by using software (Origin or Peak Fit) (Garvey et al., 2005). Gaussian functions in curve-fitting process are commonly used for the deconvolution of XRD spectra (Teeaar et al., 1987). Crystallinity can be calculated by using the equation.

$$\begin{array}{l} \text{Crystallinity degree}\left(CRI\right)\\ =\frac{Area\;of\;crystalline\;peaks}{Area\;of\;all\;peaks\;\left(\;crystalline\;+\;amorphous\right)}\times 100 \end{array}$$

### Antibacterial Activity

The antibacterial activity of TOBC and TOBC–AgSD nanocomposites against *P. aeruginosa, E. coli*, and *S. aureus* was carried out by disk diffusion method. The disk diffusion method was performed in solid agar medium LB. TOBC (control) and AgSD–TOBC nanocomposites were cut into spherical shapes (10 mm diameter) and sterilized with an ultraviolet lamp for 30 min. The sterilized samples were then placed on solid agar carefully containing bacterial solution (1 ml) in 90-mm-diameter petri dishes. The plates (petri dishes) were incubated for 24 h in an incubator at 37°C. The diameter of the inhibition zones formed was measured and recorded using a Vernier caliper (Mohseni et al., 2019).

## Results and Discussion

### Formation of TOBC–AgSD Nanocomposites

**Figure 3:**
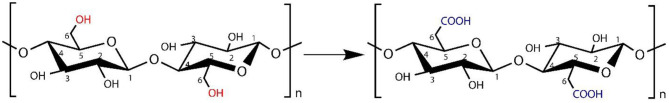
Primary hydroxyls oxidation by TEMPO/NaBr/NaClO to C6 carboxylic group of cellulose.

**Figure 4:**
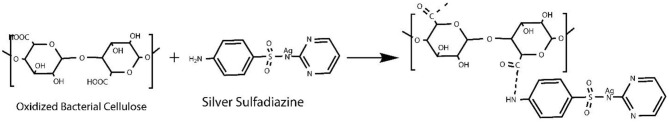
Schematic representation of TOBC–AgSD nanocomposites.

We used various concentrations of AgSD with TOBC to prepare TOBC–AgSD nanocomposites. Firstly, BC membranes, with the help of a homogenizer, were converted into BC slurry. Then, it was modified by the introduction of a carboxylic group under mild conditions using TEMPO-mediated oxidation. As the degree of oxidation increases, the hydrogen bonding between the TOBC matrix becomes stronger (Jia et al., 2019). The process is shown in Figure 3. Secondly, after oxidation, AgSD was mixed with TOBC slurry with different ratios. The proposed mechanism of the formation of TOBC–AgSD nanocomposites is shown in Figure 4.

### EDX Analysis

**Figure 5:**
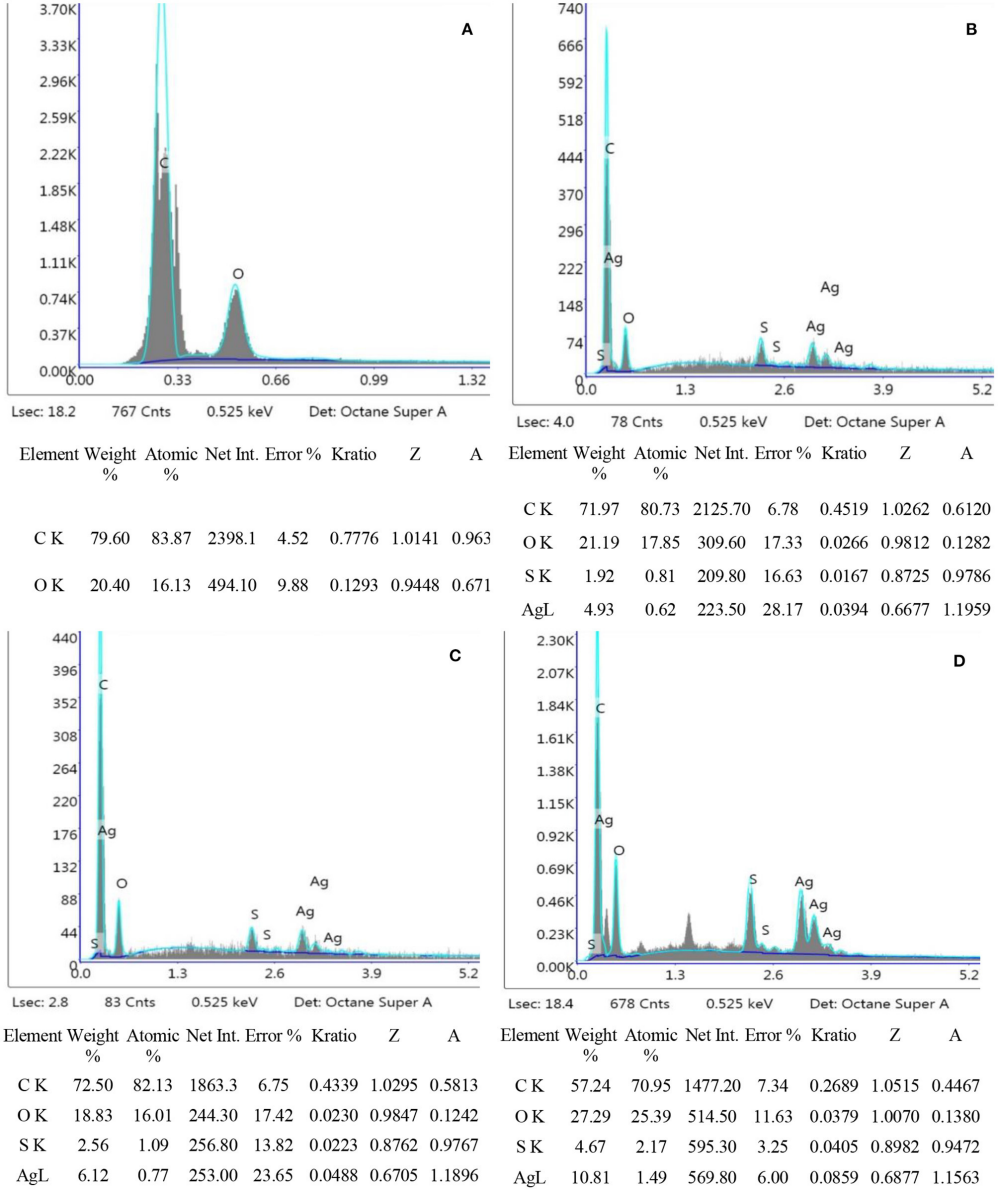
EDX analysis of TOBC **(A)**, OBCS_1_ **(B)**, OBCS_2_ **(C)**, and OBCS_3_ **(D)**.

EDX analysis was performed to quantitatively measure the content of Ag and sulfur in the nanofibers. It can be seen from Figure 5 that OBCS_1_ has lower Ag and S values than OBCS_2_. The OBCS_2_ has lower Ag and S values than Sample D, which means that Ag and S contents increase as the concentration of AgSD increases while keeping the content of the BC constant. Therefore, this is a direct control of the presence of Ag and S as active ingredients of the prepared nanofiber. In the EDX analysis, it was also observed that both Ag and S showed an upward trend in the EDX analysis.

### FTIR

**Figure 6:**
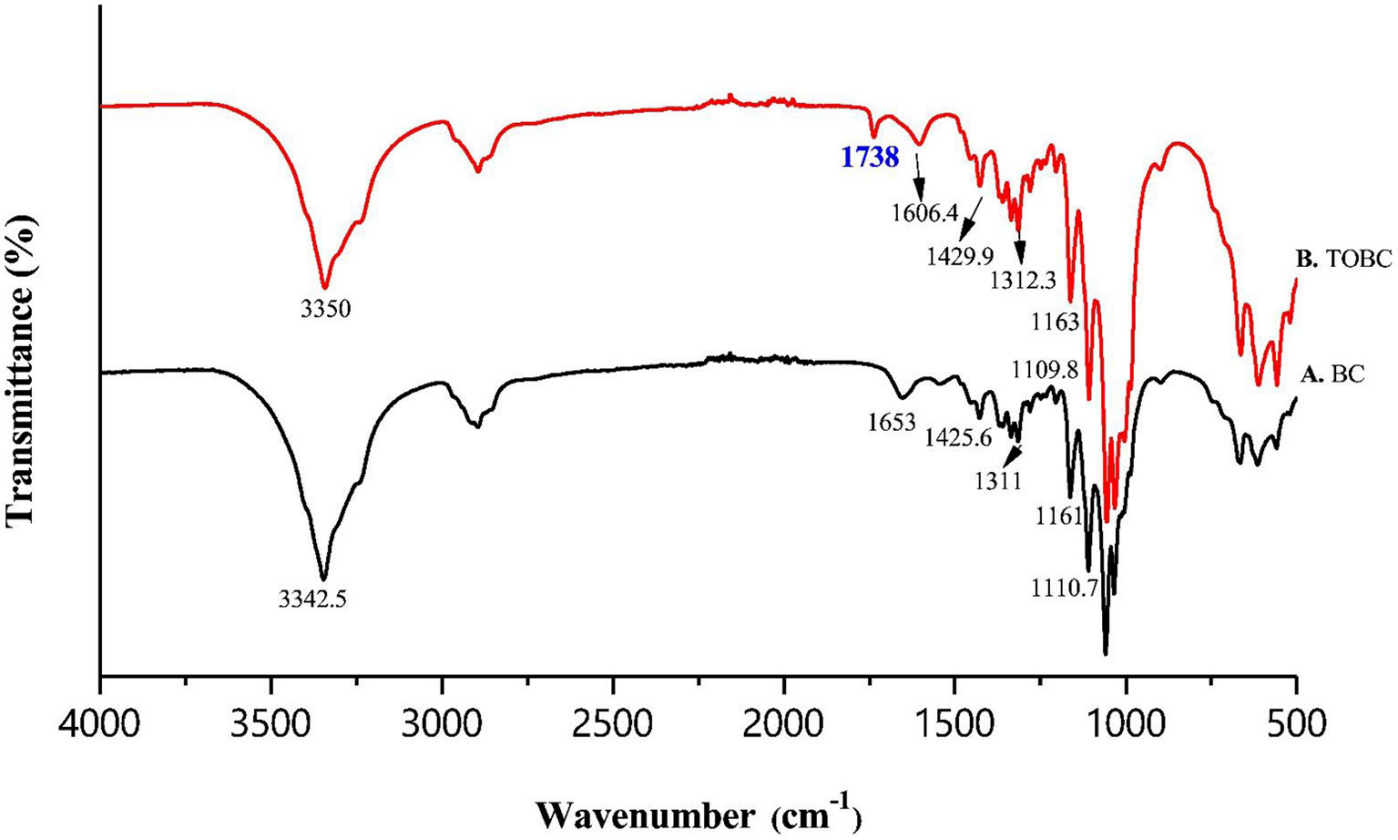
FTIR spectra of BC **(A)** and TOBC **(B)**.

**Figure 7:**
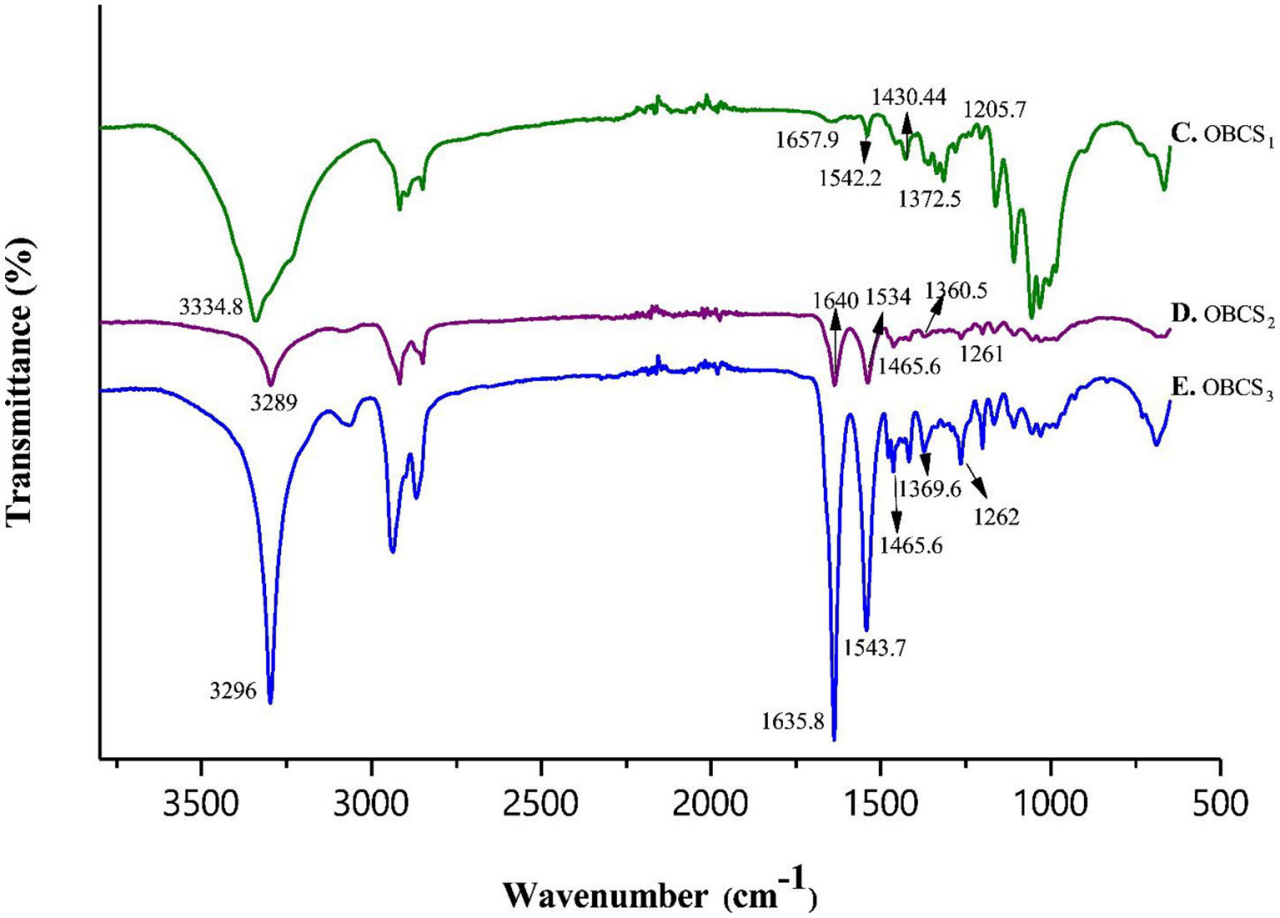
FTIR analysis of TOBC nanocomposites with different loadings of AgSD.

Fourier transform infrared spectroscopy (FTIR) analysis, was performed to assess the interaction between TOBC and AgSD. Figure 6 shows the spectra of BC and TOBC, whereas Figure 7 shows the spectra of TOBC–AgSD nanocomposites. In the case of BC and TOBC (Figures 6A,B), the FTIR spectrum obtained is typical and the dominant signals are at 3342.5–3,350 cm^−1^, which corresponds to the intra hydrogen bonding and OH stretching (Feng et al., 2012; Wasim et al., 2020). The absorbance at 1,738 cm^−1^ (Figure 6B) appeared to correspond to the carboxylic (carbonyl) group (Luo et al., 2013). The peaks at 1,163 and 1,161 cm^−1^ represent C–O asymmetric stretching, whereas the peaks at 1110.7 and 1109.8 cm^−1^ (Figures 6A,B) correspond to the C–O–C pyranose ring skeletal vibration of BC (Park et al., 2013). The characteristic bands exhibited by AgSD–TOBC nanocomposites are shown in Figure 7 (curves C, D, and E). The bands that appeared at 1205.7 cm^−1^ (C), 1,261 cm^−1^ (D), and 1,262 cm^−1^ (E) were assigned to the asymmetric stretching of SO_2_ bonds (Shao et al., 2016). The bands that appeared at 1542.2 cm^−1^ (C), 1,534 cm^−1^ (D), and 1543.7 cm^−1^ (E) can be assigned to pyrimidine skeletal vibrations due to silver (Ag^+^) ions. The obtained results were similar to the reported study (Shao et al., 2016). Moreover, the bands present at 3334.8, 3,289, and 3,296 cm^−1^ were assigned to -NH_2_ stretching bands. As the concentration of AgSD increases, the peak intensities in TOBC–AgSD nanocomposites in Figure 7 (curves C, D & E) also increase. The sample (Figure 7C) with a high concentration of AgSD exhibits a significant peak at 1543.71 cm^−1^ due to the free N–H. Similar observations have previously been reported (Fajardo et al., 2013; Zepon et al., 2014; Liu et al., 2019). From the obtained results, it can be observed that the characteristic peaks of silver sulfadiazine are present and with an increase in the peak intensity in the FTIR spectra.

### XRD Analysis

**Figure 8:**
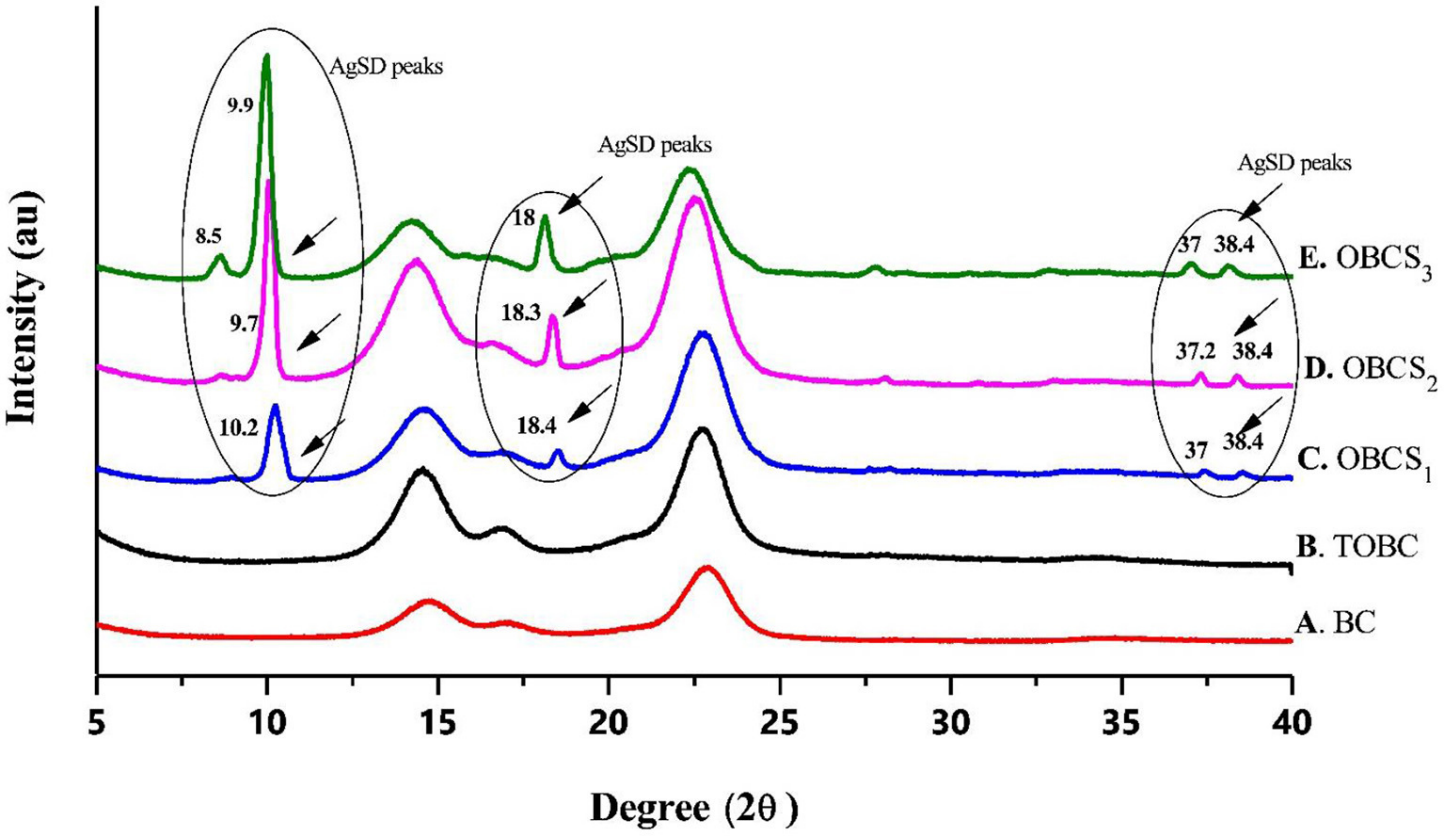
XRD analysis of BC, TOBC, and TOBC–AgSD nanocomposites with different ratios of AgSD.

Figure 8 shows the XRD analysis of BC, TOBC, and TOBC–AgSD nanocomposites (Figure 8). Two broader peaks and one small (less obvious) peak at 2θ = 14.7°, 2θ = 17°, and 2θ = 22.8° (Figure 8F) were observed and attributed to the characteristic peaks (cellulose *I*_β_ crystal) of BC. TOBC also shows these characteristics peaks (Figure 8B), which confirms the crystalline nature of TOBC. The sharp peaks at 2θ = 10.2° (C), 9.7° (D), 8.5° and 9.9° (E), 2θ = 18.4° (C), 18.3° (D), and 18°(E), and numerous small peaks at 2θ = 37° and 38.4° (C, D, and E) show and confirmed the presence of AgSD (Ullah et al., 2019). Also, after the impregnation of TOBC with AgSD, crystallinity was increased, which means that the crystalline structure of TOBC was retained (Khamrai et al., 2017). Furthermore, an increase in the ratio of AgSD to TOBC increases peak intensity. BC exhibited a crystallinity of 88%, whereas TOBC exhibited 90%, which was higher than BC. It could be due to the fact that the carboxylate generated in disordered regions combines with the hydroxyl groups to form inter-acetal linkages (Luo et al., 2013; Shao et al., 2016). The TOBC–AgSD nanocomposites showed crystallinities of 90, 92, and 93%, respectively.

### FESEM Spectroscopy

**Figure 9:**
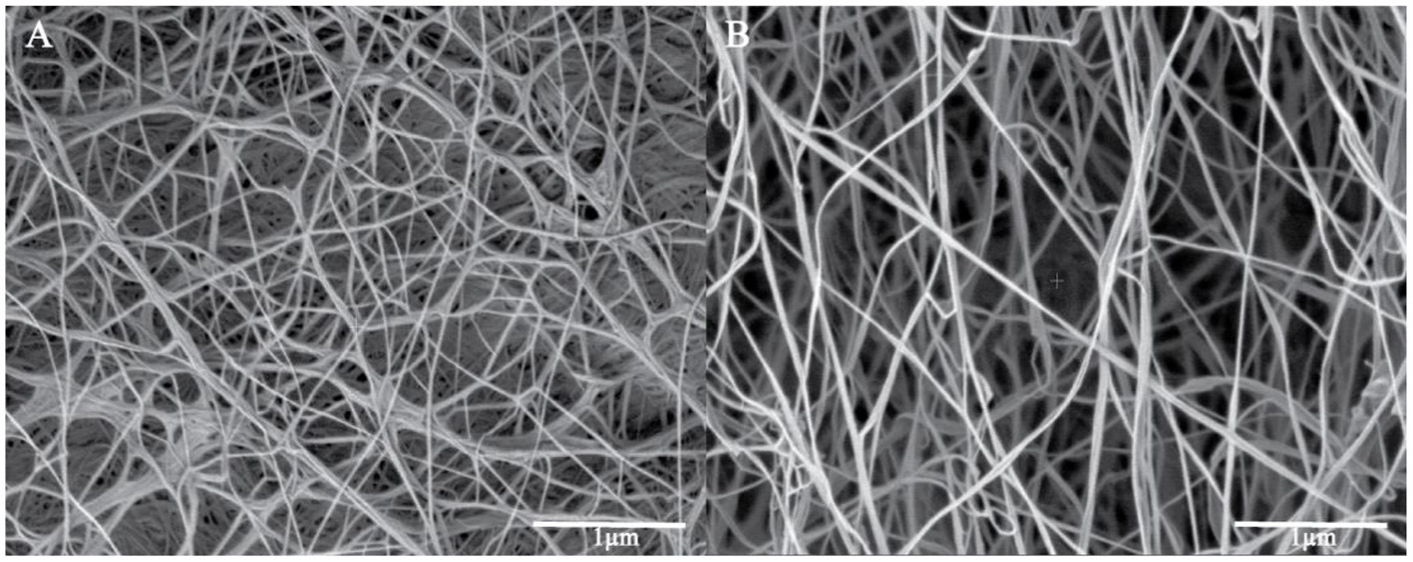
FESEM images of original BC **(A)** and TOBC **(B)**.

**Figure 10:**
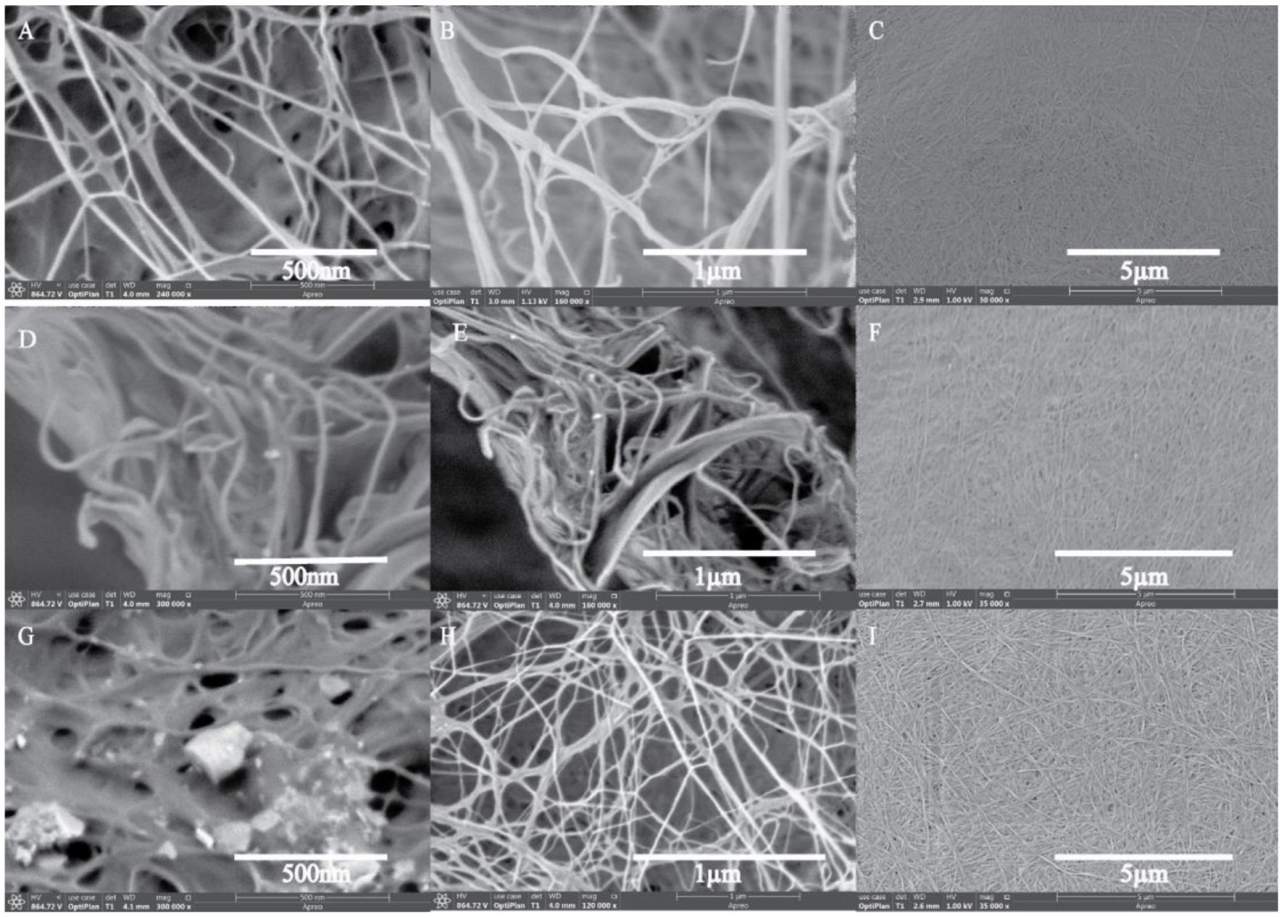
FESEM images of TOBC–AgSD nanocomposites. OBCS_1_ **(A–C)**, OBCS_2_ **(D–F)**, and OBCS_3_ **(G–I)** with different magnification.

**Figure 11:**
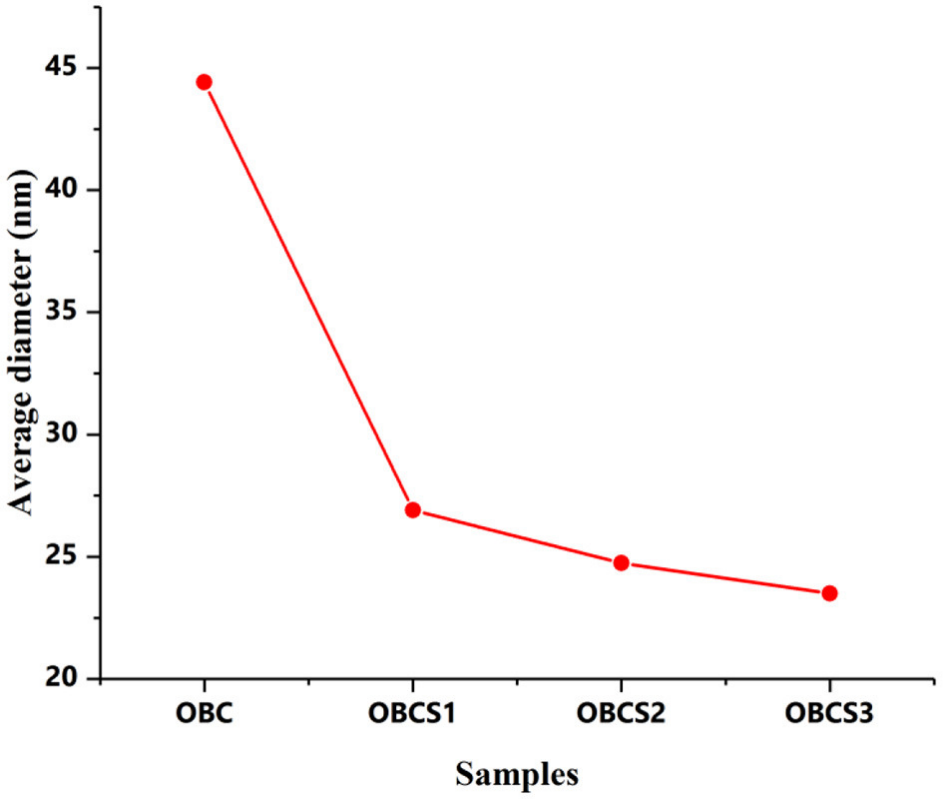
Average diameter distribution of TOBC and AgSD–TOBC nanocomposites.

Surface morphological studies of BC and TOBC (Figure 9) and TOBC–AgSD nanocomposites (Figure 10) were studied by FESEM. The results show that TEMPO oxidation did not change the morphology, i.e., the crystalline structure of BC is retained after the oxidation reaction, indicating the selective oxidation of the primary hydroxyl group on the surface of BC (Lu et al., 2014). TOBC and TOBC–AgSD nanocomposites exhibit a highly porous structure having interconnected pores, which is consistent with the other reported study (Shi et al., 2014). A denser network structure of AgSD particles with a significant dispersion in the TOBC matrix is shown in Figures 10C,F,I. An increase in the ratio of AgSD to TOBC results in the dense network structure, i.e., Figure 10G. However, for TOBC–AgSD nanocomposites with higher AgSD percentage (Figures 10G,H), larger AgSD particles accumulated in the matrix. This is because of the higher percentage of AgSD or loading of the AgSD, which leads to the overlapping with each other within the TOBC matrix. AgSd particles appear as white spots (Figure 10). In Figure 11, the average diameter graph of TOBC-AgSd nanofibers is displayed. The change in the diameter of nanofiber was observed with the addition of AgSD into the TOBC. The average of 50 nanofibers from each sample was selected and analyzed using ImageJ software. The average diameters of TOBC, OBCS_1_, OBCS_2_, and OBCS_3_ nanocomposites were 44, 27, 24, and 23 nm. The diameter of the TOBC nanofiber was nearly unchanged by the TEMPO oxidation as BC fibers consist of 50–100 nm, whereas the average diameters of the AgSD particles were 11, 13, and 17 nm (Wu et al., 2018). Usually, with the addition of nanoparticles, there is an increase in the diameter of nanocomposites. However, in this case, the results were opposite. This could be due to the strong bonding between TOBC and AgSD nanoparticles as the sonicated AgSD particles were more uniform to permeate easily in the BC fibril network (Luan et al., 2012). Similar results were also observed with the previous reported study (Khan et al., 2019; Ullah et al., 2019).

### Antibacterial Activity

**Figure 12:**
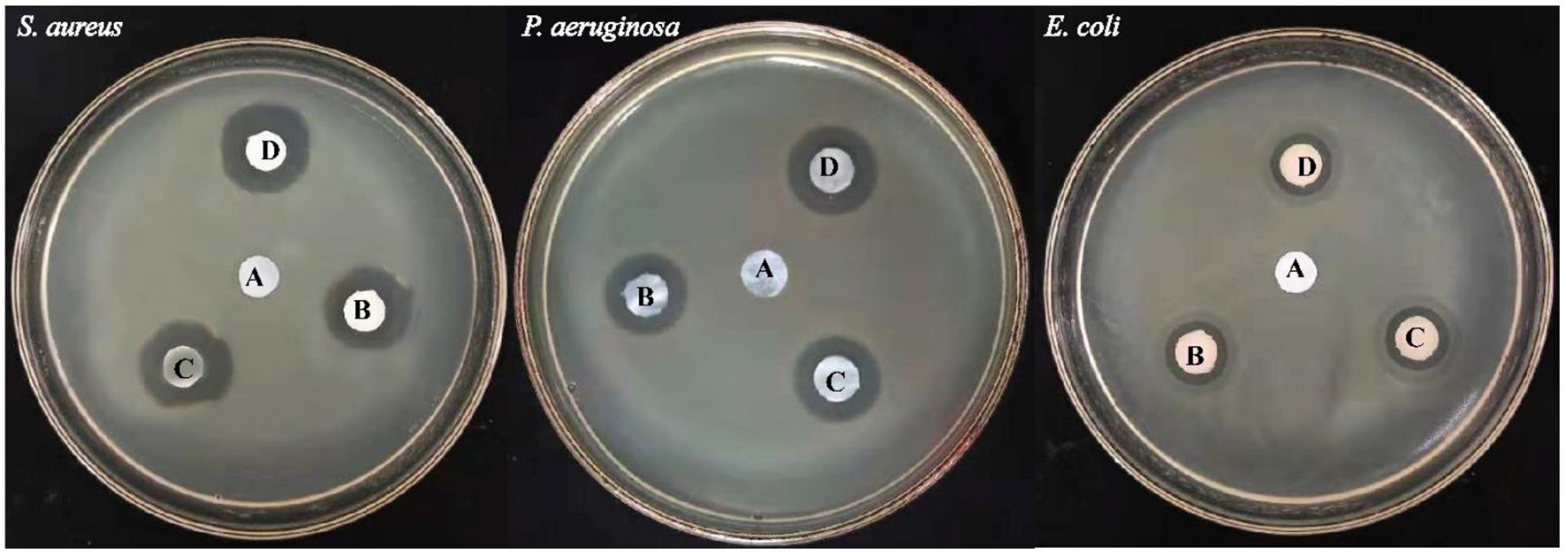
Illustrating the inhibition zones for **(A)** TOBC, **(B)** OBCS_1_, **(C)** OBCS_2_, and **(D)** OBCS_3_ by the disc diffusion method.

*E. coli, P. aeruginosa* (both Gram-negative bacteria), and *S. aureus* (Gram-positive bacteria) were selected for antibacterial testing because they are usually the main cause of infection during the healing of infection (Jo et al., 2012; Fajardo et al., 2013). After 12 h, zone of inhibition was measured to check the antibacterial activity around the sample as shown in Figure 12 using different concentrations of TOBC–AgSD. TOBC is used as a control, and no inhibition zone was observed around TOBC, which means that it does not possess any antibacterial activity against *P. aeruginosa, E. coli*, and *S. aureus*. On the other hand, due to the presence of AgSD, significant inhibition zone areas around other samples containing TOBC–AgSD confirmed their antibacterial properties as TOBC membrane is used as a matrix (Table 1). As the concentration of AgSD compound increases, there is an increase in the inhibition zone that is consistent with the reported literature (Mi et al., 2002). The zone of inhibition depends on the concentration of the AgSD. All samples exhibited excellent antibacterial efficacy, but the sample with 0.3% AgSD exhibited excellent antibacterial activity, which was also confirmed from the EDX and showed consistent results for the used bacteria. The amount of AgSD in the sample was found to increase and was very important. According to Laura et al. (2013), silver ion has major antibacterial activity against several bacteria, whereas sulfadiazine exhibits bacteriostatic properties.

**Table 1:** The inhibition zones of TOBC–AgSD nanocomposites.

**Samples**	**Inhibition zone (mm)**		
	***P. aeruginosa***	***S. aureus***	***E. coli***
TOBC (control)	00	00	00
OBCS_1_	19.11 ± 0.5	19.76 ± 0.4	12.29 ± 0.5
OBCS_2_	19.49 ± 0.4	18.60 ± 0.6	13.36 ± 0.4
OBCS_3_	21.25 ± 0.6	18.99 ± 0.5	13.81 ± 0.6

Perhaps, the difference in diameter in the zone of inhibition will be due to the difference of bacteria and their susceptibility to the prepared nanofibers. So, the results of this study are consistent with other studies especially on the bactericidal effects of AgSD (Fajardo et al., 2013).

This study clearly shows that TOBC–AgSD compounds have excellent antibacterial activity against Gram-negative and -positive bacteria; combined with all beneficial qualities, the prepared TOBC–AgSD compound is a good antibacterial material for wound dressings and also in other biomedical applications.

## Conclusion

In summary, TOBC–AgSD nanocomposites were prepared by TEMPO-mediated oxidation. TEMPO oxidation enhances the reactivity of BC. Another advantage is retaining the structure of BC after the structure modification. TEMPO oxidation pertains to mechanical properties and reactivity to BC, while silver sulfadiazine (AgSD) makes it antibacterial. To check antibacterial activity, zone of inhibition test was performed against *P. aeruginosa, S. aureus*, and *E. coli*. OBCS_3_ showed good antibacterial activity, which showed that AgSD concentrations by weight are an effective way to significantly increase antibacterial activity. In addition, the results obtained from FTIR and XRD indicated that TEMPO oxidation retains the mechanical properties and reactivity of BC, while silver sulfadiazine (AgSD) makes it antibacterial. XRD results also showed that crystallinity increases with TEMPO oxidation of BC. The results also show the potential new antibacterial applications of AgSD–TOBC membranes. Altogether, our results suggest that it could be a promising candidate for biomedical applications especially in wound dressing, tissue engineering, and BC scaffold. However, human clinical trials and studies are required to use the potential medical/pharmaceutical interest of TOBC–AgSD nanocomposites.

## Data Availability Statement

The original contributions presented in the study are included in the article/supplementary material, further inquiries can be directed to the corresponding author.

## Author Contributions

CZ conceived the project, supervised the research, and wrote the manuscript. SK and X-TQ designed and performed the experiments, analyzed the results, wrote the manuscript, and prepared the figures. SK performed the characterization and wrote the manuscript. Y-YX and X-TQ assisted in analytical studies. CZ, S-RJ, and FW assisted in characterization analysis and manuscript writing. All authors read and approved the manuscript.

## Conflict of Interest

The authors declare that the research was conducted in the absence of any commercial or financial relationships that could be construed as a potential conflict of interest.
